# Evidence of a predictive coding hierarchy in the human brain listening to speech

**DOI:** 10.1038/s41562-022-01516-2

**Published:** 2023-03-02

**Authors:** Charlotte Caucheteux, Alexandre Gramfort, Jean-Rémi King

**Affiliations:** 1 Meta AI, Paris, France; 2 Université Paris-Saclay, Inria, Commissariat à l’Énergie Atomique et aux Énergies Alternatives, Paris, France; 3 Laboratoire des systèmes perceptifs, Département d’études cognitives, École normale supérieure, PSL University, CNRS, Paris, France

**Keywords:** Language, Computer science, Computational neuroscience

## Abstract

Considerable progress has recently been made in natural language processing: deep learning algorithms are increasingly able to generate, summarize, translate and classify texts. Yet, these language models still fail to match the language abilities of humans. Predictive coding theory offers a tentative explanation to this discrepancy: while language models are optimized to predict nearby words, the human brain would continuously predict a hierarchy of representations that spans multiple timescales. To test this hypothesis, we analysed the functional magnetic resonance imaging brain signals of 304 participants listening to short stories. First, we confirmed that the activations of modern language models linearly map onto the brain responses to speech. Second, we showed that enhancing these algorithms with predictions that span multiple timescales improves this brain mapping. Finally, we showed that these predictions are organized hierarchically: frontoparietal cortices predict higher-level, longer-range and more contextual representations than temporal cortices. Overall, these results strengthen the role of hierarchical predictive coding in language processing and illustrate how the synergy between neuroscience and artificial intelligence can unravel the computational bases of human cognition.

## Abstract (web summary)

Current machine learning language algorithms make adjacent word-level predictions. In this work, Caucheteux et al. show that the human brain probably uses long-range and hierarchical predictions, taking into account up to eight possible words into the future.

## Main

In less than three years, deep learning has made considerable progress in text generation, translation and completion^1–4^ thanks to algorithms trained with a simple objective: predicting words from their nearby context. Remarkably, the activations of these models have been shown to linearly map onto human brain responses to speech and text^5–12^. Additionally, this mapping primarily depends on the algorithms’ ability to predict future words^7,8^, hence suggesting that this objective suffices to make them converge to brain-like computations.

Yet, a gap persists between humans and these algorithms: in spite of considerable training data, current language models are challenged by long story generation, summarization and coherent dialogue and information retrieval^13–17^; they fail to capture several syntactic constructs and semantics properties^18–22^ and their linguistic understanding is superficial^19,21–24^. For instance, they tend to incorrectly assign the verb to the subject in nested phrases like ‘the keys that the man holds ARE here’^20^. Similarly, when text generation is optimized on next-word prediction only, deep language models generate bland, incoherent sequences or get stuck in repetitive loops^13^.

**Fig. 1:**
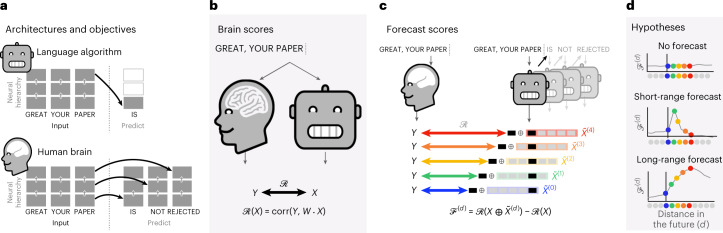
Experimental approach. **a**, Deep language algorithms are typically trained to predict words from their close contexts. Unlike these algorithms, the brain makes, according to predictive coding theory, (1) long-range and (2) hierarchical predictions. **b**, To test this hypothesis, we first extracted the fMRI signals of 304 individuals each listening to ≈26 min of short stories (*Y*) as well as the activations of a deep language algorithm (*X*) input with the same stories. We then quantified the similarity between *X* and *Y* with a ‘brain score’: a Pearson correlation ${{{\mathcal{R}}}}$ after an optimal linear projection *W* (Methods). **c**, To test whether adding representations of future words (or predicted words; Supplementary Fig. 4) improves this correlation, we concatenated (⊕) the network’s activations (*X*, depicted here as a black rectangle) to the activations of a ‘forecast window’ ($\tilde{X}$, depicted here as a coloured rectangle). We used PCA to reduce the dimensionality of the forecast window down to the dimensionality of *X*. Finally, ${{{\mathcal{F}}}}$ quantifies the gain of brain score obtained by enhancing the activations of the language algorithm to this forecast window. We repeated this analysis with variably distant windows (*d*, Methods). **d**, Top, a flat forecast score across distances indicates that forecast representations do not make the algorithm more similar to the brain. Bottom, by contrast, a forecast score peaking at *d* > 1 would indicate that the model lacks brain-like forecast. The peak of ${{{{\mathcal{F}}}}}^{d}$ indicates how far off in the future the algorithm would need to forecast representations to be most similar to the brain.

Predictive coding theory^25–27^ offers a potential explanation to these shortcomings; while deep language models are mostly tuned to predict the very next word, this framework suggests that the human brain makes predictions over multiple timescales and levels of representations across the cortical hierarchy^28,29^ (Fig. 1a).

Previous work already evidenced speech predictions in the brain by correlating word or phonetic surprisal, that is, the extent to which a word or phone is expected, with functional magnetic resonance imaging (fMRI)^30–33^, electroencephalography^34–36^, magnetoencephalography^37^ and electrocorticography^11,38^. However, such surprisal estimates derive from models trained to predict the very next word or phoneme and reduce down their output to a single number, that is, the probability of the next token. Consequently, the nature of the predicted representations and their temporal scope are largely unknown.

In this study, we address these issues by analysing the brain signals of 304 individuals listening to short stories while their brain activity is recorded with fMRI^39^. After confirming that deep language algorithms linearly map onto brain activity^6,8,40^, we show that enhancing these models with long-range and multi-level predictions improves such brain mapping. Critically, and in line with predictive coding theory, our results reveal a hierarchical organization of language predictions in the cortex, in which the highest areas predict the most distant and highest-level representations.

## Results

### Deep language models map onto brain activity

First, we quantified the similarity between deep language models and the brain, when these two systems are inputted with the same stories. For this, we used the Narratives dataset^39^ and analysed the fMRI of 304 individuals listening to short stories (27 stories ranging from 7 to 56 min; 4.6 h of unique stimulus in total, 26 min on average per participant, from 7 to 99 min). We then fitted, for each voxel and each individual independently, a linear ridge regression to predict the fMRI signals from the activations of several deep language models. Finally, we computed the corresponding ‘brain scores’ using held-out data, that is, the voxel-wise correlation between the fMRI signals and the predictions of the ridge regression input with the activations of a given language model (Fig. 1b). For clarity, we first focused on the activations of the eighth layer of Generative Pre-trained Transformer 2 (GPT-2), a 12-layer causal deep neural network provided by HuggingFace^2^ because it best predicts brain activity^7,8^.

**Fig. 2:**
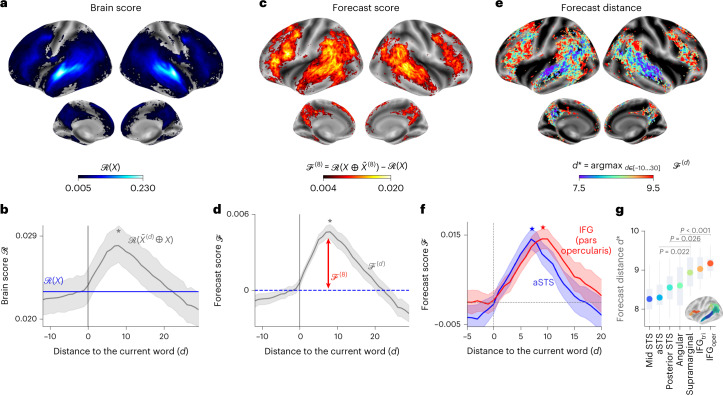
Isolating language predictions and their temporal scope in the human brain. **a**, The ‘brain score’ (${{{\mathcal{R}}}}$; Fig. 1b and Methods), obtained with GPT-2, for each individual and each voxel, here averaged across individuals (*n* = 304). Only the voxels with significant brain scores are colour-coded. **b**, Average (across voxels) brain scores obtained with GPT-2 with (grey) or without (blue) forecast representations. The average brain score peaks at d^*^ = 8 (grey star). **c**, For each voxel, the average (across individuals) ‘forecast score’ ${{{{\mathcal{F}}}}}^{d}$, that is*,* the gain in brain score when concatenating the activations of GPT-2 with a forecast window ${\tilde{X}}^{(8)}$ is shown. Only the voxels with significant forecast scores are colour-coded. **d**, Average (across voxels) forecast scores for different distance *d*. **e**, Distance that maximizes ${{{{\mathcal{F}}}}}^{d}$, computed for each individual and each voxel and denoted d^*^. This ‘forecast distance’ reveals the regions associated with short- and long-range forecasts. Regions in red and blue are associated with long-range and short-range forecasts, respectively. We only display the voxels with a significant average peak (${{{{\mathcal{F}}}}}^{{d}^{* }}-{{{{\mathcal{F}}}}}^{0},{d}^{* }=\,8$; Methods). **f**, Forecast score within two regions of interest. For each region, we report the average forecast scores of individuals with a representative peak (individuals whose peak belongs to the 45–55 percentiles of all peaks, *n* = 30 individuals). **g**, Forecast distance of seven regions of interest, computed for each voxel of each individual and then averaged within the selected brain regions. For all panels, we report the average effect across individuals (*n* = 304), with the 95% CIs across individuals (**b**,**d**,**f**). *P* values were assessed with a two-sided Wilcoxon signed-rank test across individuals. In **a**,**c**,**e**, *P* values were corrected for multiple comparisons across voxels using the FDR and brain maps are thresholded at *P* < 0.01. The boxplot in **g** summarizes the distribution of the effect obtained on ten distinct and random subdivisions of the dataset.

In line with previous studies^5,7,40,41^, the activations of GPT-2 accurately map onto a distributed and bilateral set of brain areas. Brain scores peaked in the auditory cortex and in the anterior temporal and superior temporal areas (Fig. 2a, Supplementary Fig. 1, Supplementary Note 1 and Supplementary Tables 1–3). The effect sizes of these brain scores are in line with previous work^7,42,43^: for instance, the highest brain scores (*R* = 0.23 in the superior temporal sulcus (Fig. 2a)) represent 60% of the maximum explainable signal, as assessed with a noise ceiling analysis (Methods). Supplementary Note 2 and Supplementary Fig. 2 show that, on average, similar brain scores are achieved with other state-of-the-art language models and Supplementary Fig. 3 shows that auditory regions can be further improved with lower-level speech representations. As expected, the brain score of word rate (Supplementary Fig. 3), noise ceiling (Methods) and GPT-2 (Fig. 2a) all peak in the language network^44^. Overall, these results confirm that deep language models linearly map onto brain responses to spoken stories.

### Isolating long-range predictions in the brain

Next, we tested whether enhancing the activations of language models with long-range predictions leads to higher brain scores (Fig. 1c,d). Specifically, for each word, we concatenated (1) the model activations of the present word (denoted *X*) and (2) a ‘forecast window’ (denoted ${\tilde{X}}^{(d)}$), consisting of the embeddings of future words and parameterized by a temporal distance *d* and width of *w* = 7 words (see Supplementary Fig. 4 for the growing window analysis). While the width is the number of concatenated words, *d* corresponds to the distance between the current word and the last word of the window. For instance, ${\tilde{X}}^{(10)}$ is the concatenation of words at distances 4, 5 and up to 10 from the current word, and ${\tilde{X}}^{(8)}$ is the concatenation of words at distances 2, 3 and up to 8 from the current word. For each distance *d*, we computed the ‘forecast score’ (denoted ${{{{\mathcal{F}}}}}^{d}$) by comparing the brain scores obtained with and without the forecast representations (Fig. 2b).

Our results show that ${{{\mathcal{F}}}}$ is maximal for a distance of *d* = 8 words and peaks in the areas typically associated with language processing (Fig. 2b–d). For comparison, there are 2.54 words per second on average in the stimuli. Thus, 8 words correspond to 3.15 s of audio (the time of two successive fMRI scans). These forecast scores are bilaterally distributed in the brain, except for the inferior-frontal and supramarginal gyri (*P* < 0.001 in the pars opercularis and supramarginal, using a two-sided pairwise Wilcoxon rank-sum test between the left and right hemispheres, after correcting for multiple comparisons (Methods)).

Supplementary analyses confirm that (1) each future word from word zero to ten significantly contributes to the forecast effect, (2) forecast representations are best captured with a window size of around 8 words, (3) random forecast representations do not improve brain scores and (4) using the words generated by GPT-2 instead of the true future words achieves lower but similar results (Supplementary Notes 3–5 and Supplementary Figs. 4–6).

Together, these results reveal long-range forecast representations in the brain representing a 23% (±9% across individuals) improvement in brain scores (Fig. 2a,b).

### The time range of predictions varies along the brain hierarchy

Both anatomical and functional studies have shown that the cortex is organized as a hierarchy^28,45^: for example, low-level acoustics, phonemes and semantics are primarily encoded in Heschl’s gyrus, the superior temporal gyrus and the associative cortices of the frontal, temporal and parietal lobes, respectively^42,46–49^.

Do the different levels of this cortical hierarchy predict the same time window? To address this issue, we estimated the peak of the forecast score of each voxel and denoted d^*^ the corresponding distance. The results show that the prefrontal area forecast, on average, is further off in the future than temporal areas (Fig. 2e). For instance, d^*^ in the inferior temporal gyrus (IFG) is higher than in the anterior superior temporal sulcus (aSTS) (Δd^*^ = 0.9 ± 0.2, *P* < 0.001; Fig. 2f,g).

The variation of optimal forecast distance along the temporo-parietal-frontal axis is largely symmetric across the two hemispheres (Supplementary Fig. 1).

### Predictions are increasingly contextual along the hierarchy

What is the nature of these predictive representations? To address this issue, we assessed whether the forecast score relates to (1) low or high as well as (2) syntactic or semantic representations. To this aim, we computed the forecast scores as in Fig. 1c but varied the layer used from GPT-2. Then, we identified k^*^ for each voxel, that is, the depth that maximizes the forecast scores (Methods). We considered that the deep layers of language algorithms encode higher-level and more contextualized representations than their first layers^50,51^.

**Fig. 3:**
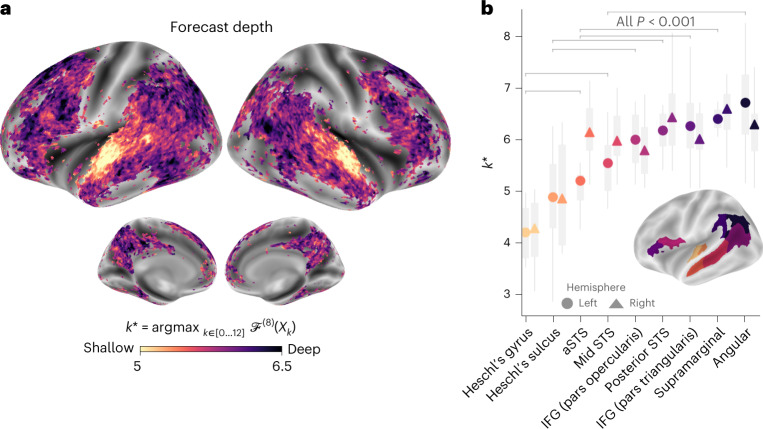
Organization of hierarchical predictions in the brain. **a**, Depth of the representation that maximizes the forecast score in the brain, denoted k^*^. Forecast scores were computed for each depth, individual and voxel, at a fixed distance of d^*^ = 8 and averaged across individuals. We computed the optimal depth for each individual and voxel and plotted the average forecast depth across individuals. Dark regions are best accounted for by deep forecasts, while light regions are best accounted for by shallow forecasts. Only significant voxels are colour-coded as in Fig. 2c). **b**, Same as **a** but with k^*^ averaged across the voxels of nine regions of interest, in the left (circle) and right (triangle) hemispheres. Scores were averaged across individuals (*n* = 304) and the boxplot summarizes the distribution of the effect obtained on ten distinct and random subdivisions of the dataset. Pairwise significance between regions was assessed using a two-sided Wilcoxon rank-sum test on the left hemisphere’s scores (the grey bars indicate *P* < 0.001).

Our results showed that the optimal forecast depth varies along the expected cortical hierarchy (Fig. 3a). Specifically, associative cortices are best modelled with deeper forecasts (k^*^ > 6) than low-level language areas (for example, k^*^ < 6 in Heschl’s gyri/sulci, aSTS; Fig. 3a,b). The difference between regions, while small on average, was highly significant across individuals (for example, between the angular and Heschl’s gyri: Δk^*^ = 2.5 ± 0.3, *P* < 0.001) and observed in both the left and right hemispheres (Fig. 3b).

Together, these results suggest that the long-range predictions of frontoparietal cortices are more contextualized and of higher level than the short-term predictions of low-level brain regions.

### Syntactic and semantic predictions show different time ranges

**Fig. 4:**
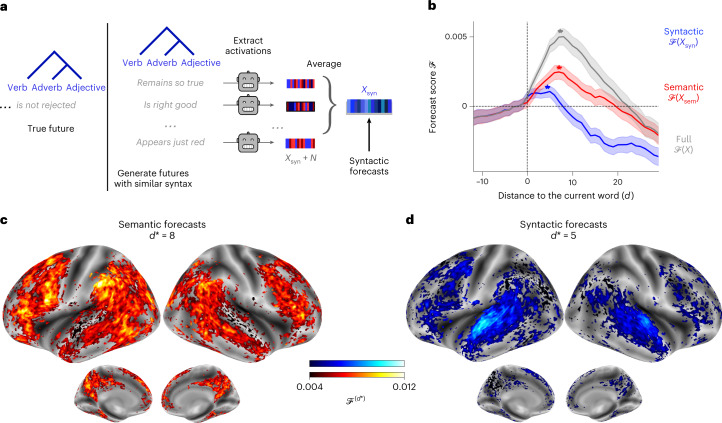
Factorizing syntactic and semantic predictions in the brain. **a**, Method to extract syntactic and semantic forecast representations, adapted from Caucheteux et al.^40^. For each word and its context (for example, ‘Great, your *paper* ... ’, we generated ten possible futures with the same syntax as the original sentence (part of speech and dependency tree) but randomly sampled semantics (for example, ‘... remains so true’, ‘... appears so small’). Then, we extracted the corresponding GPT-2 activations (layer eight). Finally, we averaged the activations across the ten futures. This method allowed us to extract the syntactic component common to the ten futures, denoted *X*_syn_. The semantic component was defined as the residuals of syntax in the full activations; *X*_sem_ = *X* − *X*_syn_. We built the syntactic and semantic forecast windows by concatenating the syntactic and semantic components of seven consecutive future words, respectively (Methods). **b**, Syntactic (blue) and semantic (red) forecast scores, on average across all voxels, as in Fig. 2c. Scores were averaged across individuals; the shaded regions indicate the 95% CIs across individuals (*n* = 304). The average peaks across individuals are indicated with a star. **c**, Semantic forecast scores for each voxel, averaged across individuals and at d^*^ = 8, the distance that maximizes the semantic forecast scores in **b**. Only significant voxels are displayed as in Fig. 2c. **d**, Same as **c** for syntactic forecast scores and d^*^ = 5.

To factorize forecast representations into syntactic and semantic components, we applied a method introduced in Caucheteux et al.^40^ and proceeded as follows: for each word and its preceding context, we generated ten possible futures, which matches the syntax of the true future words. We chose *k* = 10 possible futures following^40^. For each of these possible futures, we extracted the corresponding GPT-2 activations and averaged them across the ten possible futures (Fig. 4a and Methods). This method allowed us to decompose the activations of a given language model *X* into syntactic (the average vector, denoted *X*_syn_) and semantic components (the residuals, *X*_sem_ = *X* − *X*_syn_) (Methods). Once the syntactic and semantic forecast windows were built, we computed the corresponding forecast scores (Methods).

The results show that semantic forecasts are long range (d^*^ = 8) and involve a distributed network peaking in the frontal and parietal lobes. By contrast, syntactic forecasts (Fig. 4b) are relatively short range (d^*^ = 5) and localized in the superior temporal and left frontal areas (Fig. 4c,d). Note that the syntactic model without a forecast window (which has a lower dimensionality) performs better than the syntactic model with a distant forecast window. Such diminished scores can occur when there is no added information in the extra dimension of the regression because of the infamous curse of dimensionality^52^. This suggests that a long-range syntactic forecast is not detectable in the present dataset.

Overall, these results reveal multiple levels of predictions in the brain in which the superior temporal cortex predominantly predicts short-term, shallow and syntactic representations whereas the inferior-frontal and parietal areas predominantly predict long-term, contextual, high-level and semantic representations.

### Adapting GPT-2 into a predictive coding architecture

**Fig. 5:**
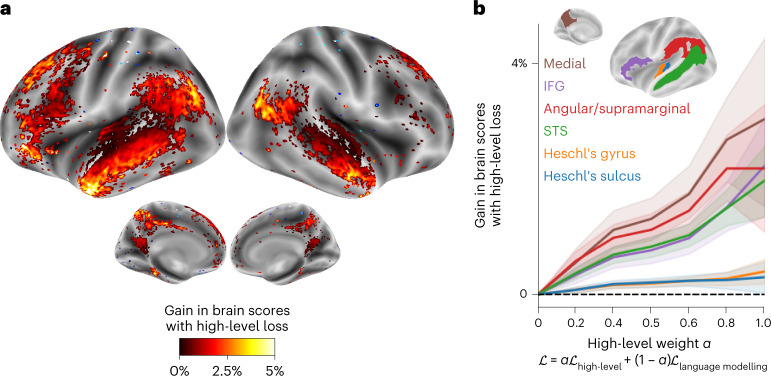
Gain in brain score when fine-tuning GPT-2 with a mixture of language modelling and high-level prediction. **a**, Gain in brain scores between GPT-2 fine-tuned with language modelling plus high-level prediction (for *α*_high level_ = 0.5) and GPT-2 fine-tuned with language modelling alone. Only the voxels with a significant gain are displayed (*P* < 0.05 with a two-sided Wilcoxon rank-sum test after FDR correction for multiple comparisons). **b**, Brain score gain as a function of the high-level weight *α* in the loss (equation (8)), from full language modelling (left, *α* = 0) to full high-level prediction (right, *α* = 1). Gains were averaged across voxels within six regions of interests (see Methods for the parcellation and Supplementary Fig. 7 for the other regions in the brain). Scores were averaged across individuals and we display the 95% CIs across individuals (*n* = 304).

These results show that concatenating present and future word representations of GPT-2 leads to a better modelling of brain activity, especially in frontoparietal areas (Fig. 2). Does fine-tuning GPT-2 to predict longer-range, more contextual and higher-level representations improve brain mapping in such regions? To answer this question, we fine-tuned GPT-2 on Wikipedia, not only using language modelling (that is, predicting the next word), but also a high-level and long-range objective (that is, predicting high-level representations of far-off words). Specifically, the high-level objective is to predict layer 8 of the pretrained GPT-2 model, of word t + 8 (Methods). The results show that GPT-2 fine-tuned with high-level and long-range modelling best accounts for frontoparietal responses (Fig. 5, >2% gain in the IFG and angular/supramarginal gyri on average, all *P* < 0.001). On the other hand, auditory areas and lower-level brain regions do not significantly benefit from such a high-level objective (Fig. 5 and Supplementary Fig. 7). These results further strengthen the role of frontoparietal areas in predicting long-range, contextual and high-level representations of language.

## Discussion

In the present study, we put specific hypotheses of predictive coding theory to the test^25–27^. While deep language algorithms are typically trained to make nearby and word-level predictions^1–3,53–55^, we assessed whether cortical hierarchy predicts multiple levels of representations, spanning multiple timescales. With this aim in mind, we compared activations of the brain to those of state-of-the-art deep language models^5–7,42,56^. We successfully validated our hypothesis on a cohort of 304 participants listening to spoken narratives^39^. Brain activity is best explained by the activations of deep language algorithms enhanced with long-range and high-level predictions. Our study provides three additional contributions.

First, the lateral, dorsolateral and inferior-frontal cortices and the supramarginal gyrus exhibited the longest forecast distances. Interestingly, these cortical regions were repeatedly linked to high-level semantics, long-term planning, attentional control, abstract thinking and other high-level executive functions^57,58^. This result echoes with previous studies showing that the integration constant of the frontoparietal cortices is larger than those of sensory and temporal areas^46,59–61^. Specifically, our findings suggest that these regions, located at the top of the language hierarchy, are not limited to passively integrating past stimuli but actively anticipate future language representations.

Second, we showed that the depth of predictive representations varies along a similar anatomical organization: low-level predictions best model the superior temporal sulcus and gyrus, while high-level predictions best model the middle temporal, parietal and frontal areas. This finding extends previous studies investigating the multiplicity of predictions underlying complex sound or speech processing^28,34,36,62^. While previous studies focused on correlating brain activity with a subset of hand-crafted and unidimensional prediction errors (for example, word or phoneme surprisal), the present analyses explored and decomposed high-dimensional predictions. More generally, our results support the idea that, unlike current language algorithms, the brain is not limited to predict word-level representations but rather predicts multiple levels of representations.

Finally, we decomposed these neural activations into syntactic and semantic representations and showed that semantic features, as opposed to syntactic ones, drive long-range forecasts. This finding strengthens the idea that while syntax may be explicitly represented in neural activity^40,63,64^, predicting high-level semantics may be at the core of long-form language processing^65,66^.

Together, these results support predictive coding theories, whereby the brain continually predicts sensory inputs, compares these predictions to the truth and updates its internal model accordingly^25,26,67^. Our study further clarifies this general framework. Not only does the brain predict sensory inputs but each region of the cortical hierarchy is organized to predict different temporal scopes and different levels of representations (Fig. 1a). However, the link between hierarchical constructs in syntax and functional hierarchy in the cortex and in the model is a major question to explore^40,51,68^.

This computational organization is at odds with current language algorithms, which are mostly trained to make adjacent and word-level predictions (Fig. 1a). Some studies investigated alternative learning rules^4,53,55,69–72^ but they did not combine both long-range and high-level predictions. We speculate that the brain architecture evidenced in this study presents at least one major benefit over its current deep learning counterparts. While future observations rapidly become indeterminate in their original format, their latent representations may remain predictable over long periods. This issue is already pervasive in speech- and image-based algorithms and has been partially bypassed with losses based on pretrained embedding^73^, contrastive learning and, more generally, joint embedding architectures^74–77^. In this study, we highlight that this issue also prevails in language models, where word sequences, but arguably not their meaning, rapidly become unpredictable. Our results suggests that predicting multiple levels of representations over multiple temporal scopes may be critical to address the indeterminate nature of such distant observations and adjust their relative confidence accordingly^78^.

Three main elements mitigate these conclusions. First, unlike temporally resolved techniques^7,11,36^, the temporal resolution of fMRI is around 1.5 s and can thus hardly be used to investigate sublexical predictions. Second, the precise representations and predictions computed in each region of the cortical hierarchy are to be characterized. This will probably require new probing techniques because the interpretation of neural representations is a major challenge to both artificial intelligence and neuroscience. Finally, the predictive coding architecture presently tested is rudimentary. A systematic generalization, scaling and evaluation of this approach on natural language processing benchmarks is necessary to demonstrate the effective utility of making models more similar to the brain.

Beyond clarifying the brain and computational bases of language, our study thus calls for systematically training algorithms to predict multiple timescales and levels of representations.

## Methods

### Notations

We denote:

- *w* as a sequence of *M* words (that is, several short stories);
- *X* as the activations of a deep language model input with *w*, of size *M* × *U*, with *U* as the dimensionality of the embeddings (for a layer of GPT-2, *U* = 768). Except if stated otherwise, we used the activations extracted from the eighth layer of a 12-layer GPT-2 model. We explicitly denote *X*_*k*_ as the activations extracted from layer *k* when using another layer;
- *Y* as the fMRI recordings elicited by *w*, of size *T* × *V*, with *T* as the number of fMRI time samples and *V* as the number of voxels;
- ${{{\mathcal{R}}}}(X)$ as the brain score of *X*;
- ${\widetilde{X}}^{(d)}$ as the forecast window containing information up to *d* words in the future. Briefly, the forecast window is the concatenation of the deep net activations of seven successive words, the last word being at a distance *d* from the current word;
- ${{{{\mathcal{F}}}}}^{(d)}(X)$ as the forecast score at distance *d*, that is, the gain in brain score when concatenating the forecast window ${\tilde{X}}^{(d)}$ to the network’s activations; ${{{{\mathcal{F}}}}}^{(d)}(X)={{{\mathcal{R}}}}(X\oplus {\tilde{X}}^{(d)})-{{{\mathcal{R}}}}(X)$;
- d^*^ as the distance maximizing the forecast score; ${d}^{* }={{{{\rm{argmax}}}}}_{d\in [-10,\ldots,30]}\,{{{{\mathcal{F}}}}}^{(d)}(X)$;
- k^*^ as the network’s depth maximizing the forecast score at a fixed distance *d* = 8; ${k}^{* }={{{{\rm{argmax}}}}}_{k\in [0,\ldots ,12]}\,{{{{\mathcal{F}}}}}^{(8)}({X}_{k})$, with *X*_*k*_ as the activations extracted from the *k*^th^ layer of GPT-2. We used *d* = 8 because it was the distance with the best forecast score on average across individuals and voxels.

### fMRI dataset

We used the brain recordings (denoted *Y*) of the Narratives dataset^39^, a publicly available dataset containing the fMRI recordings of 345 individuals listening to 27 spoken stories in English, from 7 to 56 min (4.6 h of unique stimulus in total). We use the preprocessed fMRI signals from the original dataset, without spatial smoothing (referred to as ‘afni-nosmooth’ in the repository) and sampled with TR = 1.5 s. The preprocessing steps were performed using fMRIPrep^79^; no temporal filtering was applied. The resulting preprocessing led to the analysis of cortical voxels projected onto the surface and morphed onto an ‘fsaverage’ template brain; hereafter, they are referred to as voxels for simplicity. As suggested in the original paper, some individual–story pairs were excluded because of noise, resulting in 304 individuals and 622 individual–story pairs and 4 h of unique audio material in total.

### Activations of deep language models

We compared the fMRI recordings with the activations of several pretrained deep language model inputs with the same sentences presented to the individuals. For clarity, we primarily focused on GPT-2, a high-performing causal language model trained to predict words given their previous context. GPT-2 consists of 12 Transformer modules^1,2^, each of them referred to as ‘layer’, stacked onto one non-contextual word embedding layer. We used the pretrained models from Huggingface^80^ (1.5 billion parameters trained on 8 million Web pages).

In practice, to extract the activations *X* elicited by a sequence of *M* words *w* from the *k*^th^ layer of the network, we (1) formatted the textual transcript of the sequence *w* (replacing special punctuation marks such as ‘-’ and duplicated marks ‘?.’ by dots), (2) tokenized the text using the Huggingface tokenizer, (3) inputted the network with the tokens and (4) extracted the corresponding activations from layer *k*. This resulted in a vector of size *M* × *U*, with *M* the number of words and *U* the number of units per layer (that is, *U* = 768). Given the constrained context size of the network, each word was successively inputted to the network with at most 1,024 previous tokens. For instance, while the third word’s vector was computed by inputting the network with (*w*_1_, *w*_2_, *w*_3_), the last word’s vector *w*_*M*_ was computed by inputting the network with (*w*_*M*−1,024_,…,*w*_*M*_). The alignment between the audio recordings of the stories and their textual transcripts was provided in the original Narratives database^39^.

### Brain scores

Following previous works^7,42,56^, we evaluated, for each individual *s* and voxel *v*, the mapping between (1) the fMRI activations *Y*^(*s*,*v*)^ in response to the audio stories and (2) the activations *X* of the deep network input with the textual transcripts of the same stories. To this end, we fitted a linear ridge regression *W* on a training set to predict the fMRI scans given the network’s activations. Then, we evaluated this mapping by computing the Pearson correlation between predicted and actual fMRI scans on a held-out set:

1 $${{{{\mathcal{R}}}}}^{(s,v)}:X\mapsto {{{\rm{corr}}}}\left(W\cdot X,{Y}^{(s,v)}\right)$$

with *W* as the fitted linear projection, corr as Pearson’s correlation, *X* as the activations of GPT-2 and *Y*^(*s*,*v*)^ as the fMRI scans of one individual *s* at one voxel *v*, both elicited by the same held-out stories.

In practice and following Huth et al.^42^, we modelled the slow bold response thanks to a finite impulse response (FIR) model with six delays (from 0 to 9 s, TR = 1.5 s). Still following Huth et al.^42^, we summed the model activations of the words presented within the same TR to match the sampling frequency of the fMRI and language models (Supplementary Figs. 8 and 9). Then, we estimated the linear mapping *W* with an *ℓ*_2_-penalized linear regression after standardizing the data and reducing their dimensionality (for computational reasons). We implemented scikit-learn^81^ and used a pipeline with the following steps: (1) standardization of the features (set to 0 mean with an s.d. of 1 using a StandardScaler), (2) principal component analysis (PCA) with 20 components and (3) *ℓ*_2_-penalized linear regression (RidgeCV in scikit-learn). In Supplementary Fig. 3c, we replicated the main analyses without PCA (the brain scores and forecast effect were slightly underestimated by the PCA). The regularization hyperparameter of the RidgeCV was selected with a nested leave-one-out cross-validation among ten possible values log-spaced between 10^−1^ and 10^8^ for each voxel and each training fold.

The outer cross-validation scheme, which allows for an independent performance evaluation, uses five folds obtained by splitting the fMRI time series into five contiguous chunks. The Pearson correlations averaged across the five test folds is called ‘brain score’ and denoted as ${{{{\mathcal{R}}}}}^{(s,v)}(X)$. It measures the mapping between the activation space *X* and the brain of one individual *s* at one voxel *v* in response to the same language stimulus.

In Fig. 2a,b, brain scores were computed for each (individual, voxel) pair. We then averaged brain scores across individuals (Fig. 2a) and/or voxels (Fig. 2b) depending on the analysis. For simplicity, we denote ${{{\mathcal{R}}}}(X)$ as the brain scores averaged across individuals and/or voxels.

### Forecast windows

We tested whether adding forecast representations would improve our ability to predict brain activity. To this aim, we did not modify the deep network itself but added forecast representations to the encoding model’s input, that is, the forecast window. The forecast window at distance *d*, denoted by ${\widetilde{X}}^{(d)}$, is the concatenation of the network’s activations of seven successive words, the last one being at a distance *d* from the current word. Precisely, the forecast window of a word *w*_*n*_ at a distance *d* is the concatenation of the network’s activations elicited by words *w*_*n* + *d*−6_, …, *w*_*n* + *d*_. Thus,

2 $${\widetilde{X}}^{(d)}={({X}_{{w}_{n+d-7}}\oplus \cdots \oplus {X}_{{w}_{n+d}})}_{n\in [1,\ldots ,M]}$$

with ⊕ as the concatenation operator and *M* as the number of words in the transcript *w* (Supplementary Fig. 9). Note that *d* can be negative: in that case, the forecast window only contains past information. Except if stated otherwise, the forecast window was built out of the activations *X* extracted from the eighth layer of GPT-2. In Fig. 3, the forecast window was built out of the activations *X*_*k*_ extracted from different layers *k* of GPT-2. We denoted ${\widetilde{X}}_{k}^{(d)}$ as the corresponding forecast windows. In Fig. 4, the forecast windows were built out of the syntactic (*X*_syn_) and semantic (*X*_sem_) activations of GPT-2.

### Forecast scores

For each distance *d*, individual *s* and voxel *v*, we computed the ‘forecast score’ ${{{{\mathcal{F}}}}}^{(d,s,v)}$, which is the gain in brain score when concatenating the forecast windows to the present GPT-2 activations. Thus,

3 $${{{{\mathcal{F}}}}}^{(d,s,v)}:X\mapsto {{{{\mathcal{R}}}}}^{(s,v)}(X\oplus {\widetilde{X}}^{(d)})-{{{\mathcal{R}}}}(X)$$

To match the dimensionality of *X* and $\tilde{X}$, the PCA used to compute the mapping was trained on *X* and $\tilde{X}$ separately before concatenating the two features, that is, ${{{\mathcal{F}}}}(X)={{{\mathcal{R}}}}({{{\rm{PCA}}}}(X)+{{{\rm{PCA}}}}(\tilde{X}))-{{{\mathcal{R}}}}({{{\rm{PCA}}}}(X))$.

### Forecast distance

To test whether the forecast scope varied along the cortical hierarchy, we estimated the distance maximizing the forecast score. Precisely, the optimal ‘forecast distance’ d^*^ for each individual *s* and voxel *v* was defined as:

4 $${d}_{(s,v)}^{* }={{{{\rm{argmax}}}}}_{d\in [-10,\ldots ,30]}{{{{\mathcal{F}}}}}^{(d,s,v)}(X)$$

with *X* as the activations of the language model and ${{{{\mathcal{F}}}}}^{(d,s,v)}$ as the forecast score at distance *d* for individual *s* and voxel *v* (equation (3)). The forecast distances d^*^ were then averaged across individuals and/or voxels depending on the analyses.

The present analysis is only relevant for the brain regions for which forecast scores are not flat. Indeed, computing the distance maximizing a flat curve would be misleading. Thus, in Fig. 2e, we computed the difference ${{{{\mathcal{F}}}}}^{8}-{{{{\mathcal{F}}}}}^{0}$ for each individual and voxel, assessed the significance with a Wilcoxon rank-sum test across individuals and ignored the voxels with a non-significant difference (*P* > 0.01).

### Forecast’s depth

To test whether the depth of the forecast varied along the cortical hierarchy, we computed the forecast score for different depths of representation. We replaced *X* by the activations *X*_*k*_ extracted from layer *k* of GPT-2 (*k* ∈ [0, …, 12]) in equations (3) and (4). Then, we computed the depth maximizing the forecast score, called ‘forecast depth’, and given by:

5 $${k}_{(d,s,v)}^{* }={{{{\rm{argmax}}}}}_{k\in [0,\ldots ,12]}{{{{\mathcal{F}}}}}^{(d,s,v)}({X}_{k})$$

with ${{{{\mathcal{F}}}}}^{(d,s,v)}({X}_{k})={{{{\mathcal{R}}}}}^{(s,v)}({X}_{k}\oplus {\widetilde{{X}_{k}}}^{(d)})-{{{\mathcal{R}}}}({X}_{k})$ (equation (3)). For simplicity, we studied the depth focusing on the fixed distance *d* = 8 (Fig. 3c,d), which maximizes the forecast score in Fig. 2.

### Decomposing model activations into syntactic and semantic components

To extract the syntactic and semantic components of *X*, a vector of activations in response to a story *w*, we applied a method introduced in Caucheteux et al.^40^ (Fig. 4a). For each word, (1) we generated *n* = 10 futures of the same syntax as the true future (that is, same part of speech and dependency tags as the true future) but randomly sampled semantics, (2) we computed the activations for each of the 10 possible futures and (3) we averaged the activations across the 10 futures. We used the same hyperparameter *n* = 10 as in the original paper. The method actually converges from n = 7 (Supplementary Fig. 8 in the paper). This method allows to extract the average vector *X*_syn_, which contains syntactic information but is deprived of semantic information. The semantic activations *X*_sem_ = *X* − *X*_syn_ are the residuals of syntax in the full activations *X*. In the original paper (Fig. 3), the authors checked with probing analyses that the syntactic embeddings encoded relevant syntactic information (part of speech and depth of the syntactic tree) and no longer encoded semantic information (word frequency, word embedding, semantic category).

### Syntactic and semantic forecast windows

To investigate syntactic and semantic forecasts in the brain, we built forecast windows out of the syntactic and semantic activations of GPT-2, respectively. To this aim, we first built the forecast windows out of GPT-2 activations ${\widetilde{X}}^{(d)}$. Then, we extracted the syntactic ${\widetilde{X}}_{{{{\rm{syn}}}}}^{(d)}$ and semantic ${\widetilde{X}}_{{{{\rm{sem}}}}}^{(d)}$ components of the concatenated activations, as introduced in Caucheteux et al.^40^. Finally, the syntactic forecast score is the increase in brain score when concatenating the syntactic window:

6 $${{{{\mathcal{F}}}}}_{{{{\rm{syn}}}}}^{(d)}={{{\mathcal{R}}}}(X\oplus {\widetilde{X}}_{{{{\rm{syn}}}}}^{(d)})-{{{\mathcal{R}}}}(X)$$

Similarly, the semantic forecast score is given by:

7 $${{{{\mathcal{F}}}}}_{{{{\rm{sem}}}}}^{(d)}={{{\mathcal{R}}}}(X\oplus {\widetilde{X}}_{{{{\rm{sem}}}}}^{(d)})-{{{\mathcal{R}}}}(X)$$

### Brain parcellation

We systematically implemented whole-brain analyses and computed scores for each voxel in the brain. Yet, for simplicity, we report the scores averaged across selected regions of interest in Figs. 2f,g and 3c. To this aim, we used a subdivision of the Destrieux atlas^82^. Regions with more than 500 vertices were split into smaller parts. This resulted in 142 regions per hemisphere, each containing fewer than 500 vertices.

**Table:** 

This results in 142 regions per hemisphere, each containing fewer than 500 vertices	
STG / STS	Superior temporal gyrus / sulcus
aSTS	Anterior STS
mSTS	Mid STS
pSTS	Posterior STS
Angular / Supramar	Angular / Supramarginal inferior parietal gyrus
IFG / IFS	Inferior frontal gyrus / sulcus
Tri / Op	Pars triangularis / opercularis (IFG)
Heschl G / Heschl S	Heschl gyrus / sulcus

### Statistical significance

We systematically implemented single-individual and whole-brain analyses: all metrics (brain score, forecast score, forecast distance and depth) were computed for each individual–voxel pair. We report the metrics averaged across individuals and/or voxels depending on the analysis. Statistics were computed across individuals using a two-sided Wilcoxon rank-sum test from Scipy^83^ assessing whether the metric (or the difference between two metrics) was significantly different from zero and then corrected for multiple comparisons using the false discovery rate (FDR). We report an effect as significant if *P* < 0.01. The shaded regions in Figs. 2, 4 and 5 correspond to the 95% confidence intervals (CIs) across individuals (*n* = 304). The boxplots in Figs. 2–5 summarize the distribution of the effect obtained on 10 distinct and random subdivisions of the dataset.

### Noise ceiling

The fMRI recordings are inherently noisy. To assess the amount of explainable signal, we used a ‘noise ceiling’ analysis, that is, we predicted the brain responses *Y*^(*s*)^ of each individual *s* given the responses of the other individuals to the same story $\overline{Y}$. We proceeded similarly as the brain score computation and applied the same setting as equation (1) but used the average brain signals of other individuals’ brains ${\overline{Y}}^{(s)}=\frac{1}{| {{{\mathcal{S}}}}| }{\sum }_{{s}^{{\prime} }\ne s}{Y}^{({s}^{{\prime} })}$ (of size *T* × *V*) instead of the network’s activations *X*. Precisely:

- For the brain score computation, *Y*^(*s*)^ represents the fMRI recordings of individual *s*, corresponding to all the stories individual *s* listened to while being scanned. *X* consists of the contextual embeddings of the corresponding words, summed within each TR and transformed with FIR. Thus,
  $${R}_{{{{\rm{brain}}\ {\rm{score}}}}}(s)={{{\rm{corr}}}}[{W}^{(s)}\cdot X,{Y}^{(s)}]$$
  with *X* as the GPT-2 embeddings, temporally aligned with *Y* using FIR.
- For the noise ceiling computation, *Y*^(*s*)^ is the same as for the brain score computation. *X* consists of the average fMRI recordings of the other individuals who listened to the same stories as individual *s*. *X* and *Y* have the same dimensionality and the bold delay is assumed to be comparable across individuals, so we did not apply a FIR to X. Thus,
  $${R}_{{{{\rm{noise}}\ {\rm{ceiling}}}}}(s)={{{\rm{corr}}}}[{W}^{(s)}\cdot {\overline{Y}}^{(s)},{Y}^{(s)}]$$
  with *Y*^(*s*)^ as the average fMRI of the other individuals who listened to the same story as individual *s*.

For both the brain score and noise ceiling computation, we fitted a ridge regression *W*^(*s*)^ for each individual *s*, predicting *Y*^(*s*)^ given *X*, using the same fivefold cross-validation setting. We evaluated the prediction successively on the five test folds using Pearson correlation and averaged the correlation scores across folds. This resulted in one brain score and one noise ceiling estimate per individual (and voxel). Results averaged across individuals are displayed in Supplementary Fig. 10. This score is one possible upper bound for the best brain score that can be obtained given the level of noise in the dataset.

### Fine-tuning GPT-2 with a long-range and high-level objective

Does fine-tuning GPT-2 to predict long-term, high-level and more contextualized representations increase its similarity with the brain?

To test this question, we fine-tuned GPT-2 using a mixture of language modelling loss and high-level and long-term loss. We then evaluated brain scores and test whether the high-level objective would lead to significantly higher brain scores than the language modelling objective.

#### Architecture and losses

We fine-tuned the pretrained GPT-2 model provided by Huggingface with a mixture of language modelling and high-level forecast. The mixture loss was parameterized by a hyperparameter *α* ∈ [0,1]. The total loss minimized is given by:

8 $${{{\mathcal{L}}}}={\alpha }^{{\prime} }{{{{\mathcal{L}}}}}_{\mathrm{high-level}}+(1-{\alpha }^{{\prime} }){{{{\mathcal{L}}}}}_{\mathrm{language}\ {\mathrm{modelling}}}$$

with the constraint that ${\alpha }^{{\prime} }{{{{\mathcal{L}}}}}_{\mathrm{high-level}}=\alpha (1-{\alpha }^{{\prime} }){{{{\mathcal{L}}}}}_{\mathrm{language}\ {\mathrm{modelling}}}$. Doing so, setting *α* to 0.5 means that each term of the loss contributes to 50% of the total loss. The language modelling objective predicts the next word and it is given by:

$${{{{\mathcal{L}}}}}_{\mathrm{language}\ {\mathrm{modelling}}}={{{\rm{CE}}}}\left[{h}_{\mathrm{language}\ {\mathrm{modelling}}}\circ f({x}_{t}),{x}_{t+1}\right]$$

with:

- CE as the cross-entropy loss;
- *f* as the learned fine-tuned model. *f* is initialized with the weights of pretrained GPT-2. Thus, *f* is a 12-layers Transformer network stacked onto a word embedding, each layer having a dimensionality of 768;
- $h_{{\rm{language}}\,{\rm{modelling}}}$ as the language modelling linear head on top of the last layer of *f*, from 768 to *n*_vocab_, which predicts the next word;
- *x*_*t*_ as the input tokens;
- *x*_*t* + 1_ as the input tokens shifted from one time step (the succeeding words).

The high-level objective predicts layer *k* of word at distance *d* from the current word and it is given by:

$${{{{\mathcal{L}}}}}_{\mathrm{high-level}}^{k,d}={{{\rm{CPC}}}}[{h}_{\mathrm{high-level}}\circ f({x}_{t}),{N}^{k}({x}_{t+d})]$$

where:

- *N*^*k*^ is a separate and fixed network. Here, we use the pretrained version of GPT-2 provided by Huggingface, taken at layer *k*. Its weights are fixed: they do not vary with training.
- $h_{{\rm{high}}\hbox{-}{\rm{level}}}$ is a linear head on top of the last layer of *f*, from 768 to 768, which predicts the activations of the *k*^th^ layer of the fixed network *N*^*k*^, corresponding to the word at distance *d* from the current word.
- *x* represents the inputs, *x*_*t*_ marks the current words and *x*_*t* + *d*_ marks the words at distance *d* from the current word.
- CPC is the contrastive predicting coding loss^84^.
  $${{{\rm{CPC}}}}=-{{{\rm{log}}}}\frac{{{{\rm{Exp}}}}\left[S\left({y}_{{{{\rm{predicted}}}}},{y}_{{{{\rm{true}}}},{{{\rm{positive}}}}}\right)/\tau \right]}{{\sum }_{\mathrm{negative}}{{{\rm{Exp}}}}\left[S\left({y}_{{{{\rm{predicted}}}}},{y}_{{{{\rm{true}}}},{{{\rm{negative}}}}}\right)/\tau \right]}$$
  with *S* as a similarity metric, *y*_true,negative_ as a set of negative samples and *y*_true,positive_ as a set of positive samples.

In practice, we chose to predict the hidden states at layer *k* = 8 of the future word at distance *d* = 8. We chose layer *k* = 8 and *d* = 8 because it led to the best results (Fig. 2d). To compute the CPC loss, we took *τ* = 0.1 and used the cosine similarity as similarity metric *S*. We used 2,000 negatives randomly sampled from a negative queue (of size 2,500). The negative queue was updated at each batch by adding the hidden states to the non-target words from the current batch. Such hidden states were extracted from the pretrained network at layer *k* (*N*^*k*^). For the high-level and language modelling losses to have a fixed contribution *α* and 1 − *α* over training, we updated the parameter ${\alpha }^{{\prime} }$ in equation (8) every 100 gradient steps.

#### Dataset and training

We fine-tuned GPT-2 on the already preprocessed English Wikipedia dataset (https://huggingface.co/datasets/wikipedia) consisting of 6M documents (30 GB) on 2 graphics processing units. We used the ‘Trainer’ implementation from Huggingface with the default training arguments (Adam optimizer, learning rate = 0.00005; see https://huggingface.co/docs/transformers/main_classes/trainer for the other default parameters). Because of memory constraints, we restricted the context size of GPT-2 to 256 tokens and used a batch size of 4 per device (thus, 2 × 4 × 256 = 1,024 tokens per batch and gradient updates). For stability, we fine-tune the top tier layers of the network (from layer 8 to layer 12), while the bottom layers were kept frozen. Fine-tuning the whole network with language modelling led to a significant drop in brain scores (with fixed training parameters). Losses were monitored on a separate evaluation set of 1,000 Wikipedia documents.

#### Evaluation

We fine-tuned seven GPT-2 models with different high-level weight *α*, from a loss being full language modelling (*α* = 0), half language modelling and high-level (*α* = 0.5) to full high-level (*α* = 1). During the training, we saved ≈15 model checkpoints (regularly log-spaced between 0 and 10^6^ gradient updates). For each model and step, we computed the brain scores of its concatenated layers [0,4,8,12] on the same Narratives dataset^39^. We chose to span all layers from 0 to 12 because representations could ‘move’ across layers during the fine-tuning, which could bias the results. We then averaged the brain scores across steps and assessed the gain of one network over another. In Fig. 5, we report the gain averaged across individuals when adding increasingly more high-level prediction in the loss.

### Reporting summary

Further information on research design is available in the Nature Portfolio Reporting Summary linked to this article.

## Supplementary information

Supplementary Information Supplementary Notes 1–5, Figs. 1–10 and Tables 1–3.

Reporting Summary

## Data Availability

The Narratives dataset^39^ is publicly available on OpenNeuro https://openneuro.org/datasets/ds002345/versions/1.1.4.

## Code availability

All analyses were performed using Python and scikit-learn^81^. The fMRI data were analysed with nilearn (https://nilearn.github.io/stable/index.html), mne-python^85–88^ and freesurfer (https://surfer.nmr.mgh.harvard.edu/). Deep language models were analysed using the transformers library^80^. Statistical significance was assessed using Scipy^83^.
