# Supplementary material for: Evidence of a predictive coding hierarchy in the human brain listening to speech
Source: Nat Hum Behav. 2023 Mar 2;7(3):430–41. doi: 10.1038/s41562-022-01516-2 (PMC10038805; doi:10.1038/s41562-022-01516-2)
Supplement: Supplementary file 1 — Supplementary Notes 1–5, Figs. 1–10 and Tables 1–3. [file 41562_2022_1516_MOESM1_ESM.pdf]

nature human behaviour

Article
https://doi.org/10.1038/s41562-022-01516-2

Evidence of a predictive coding hierarchy in the human brain listening to speech

In the format provided by the authors and unedited

Supplementary Information

Supplementary Note 1. Scores per region of interest.

For clarity, we report in Supplementary Figure 1 the average brain scores, forecast scores and forecast distances for each region of interest in both the left and right hemispheres. We also report scores in the less noisy voxels, for the subjects with the highest brainscore (Supplementary Table 1), their corresponding p-values computed across subjects (Table Supplementary 3) and the scores normalized by the noise ceiling (Supplementary Table 2).

Supplementary Table 1. Brain and forecast scores in language areas. Scores averaged across all voxels in the brain (Avg), across the ten percent less noisy voxels (w.r.t the noise ceiling, Top10Vox), for the ten percent subjects with the highest brainscore (Top10sub), and averaged across voxels in representative language areas (Heschl, STG, STS and IFG). The last row is the relative improvement of $R(X+\widetilde{X})$ over $R(X)$.

	Avg	Top10Vox	Top10Sub	Heschl	STG	STS	IFG
Brain score, $R(X)$	0.023	0.084	0.049	0.145	0.072	0.072	0.037
Forecast score, $F^{(8)}(X)$	0.005	0.010	0.006	0.008	0.008	0.010	0.008
Relative improvement, $\frac{F^{(8)}(X)}{R(X)}$	23%	13%	39%	5%	21%	13%	18%

Supplementary Table 2. Brain and forecast scores in language areas, with noise ceiling normalization. Same as Table 1, but, for each voxel, scores are divided by the average noise ceiling.

	Avg	Top10Vox	Top10Sub	Heschl	STG	STS	IFG
Brain score $R(X)$	17%	37%	37%	50%	32%	36%	24%
Forecast score $F^{(8)}(X)$	4%	5%	5%	3%	4%	5%	5%

Supplementary Table 3. Brain and forecast scores' significance. Same as Table 1, but we indicate the p-values computed across subjects, testing whether the scores (either $R(X)$, $R(X+\widetilde{X})$ or $F(X)$) are different from zero. We use a two-sided Wilcoxon test provided by Scipy. The p-values for the Top10Sub columns are higher because we restrict ourselves to the 10 percent less noisy subjects.

	Avg	Top10Vox	Top10Sub	Heschl	STG	STS	IFG
Brain score $R(X)$	10^{-50}	10^{-51}	10^{-6}	10^{-51}	10^{-51}	10^{-50}	10^{-45}
Forecast score $F^{(8)}(X)$	10^{-35}	10^{-37}	10^{-4}	10^{-32}	10^{-37}	10^{-32}	10^{-29}

Supplementary Note 2. Generalisation to other architectures.

The analyses in the main manuscript focus on one representative deep neural network: GPT-2 (2). Here, we replicate our results with the activations extracted from seven other transformer architectures. We only analyse *causal* models, trained to predict a word from their *previous* context. Note that XLNet is trained to predict both left and right context (71), but, here, we only input the model with left context when extracting the activations. Similarly as with GPT-2, we use the pretrained models from Huggingface (labeled 'distilgpt2', 'gpt2', 'gpt2-medium', 'gpt2-large', 'gpt2-large', 'gpt2-xl', 'transfo-xl-wt103', 'xlnet-base-cased', 'xlnet-large-cased'), based on GPT-2 (2), XLNet (71) and Transformer-XL (86) architectures, and focus on one intermediate-to-deep layer of the model ($l = \frac{2}{3} \times n_{\text{layers}}$). For each architecture, we 1) extract the activations corresponding to the subjects' stories (Methods C) 2) compute the corresponding brain scores (Methods D) and forecast scores (Methods F) for each voxel, subject, and forecast distance. As displayed in Supplementary Figure 2, the seven architectures accurately map onto brain activity (Supplementary Figure 2**a**), and the mapping is improved when adding information about around eight words in the future (Supplementary Figure 2**b**). The mapping is also improved when adding representations of words automatically generated by GPT-2 instead of the true future words (we use sampling methods to generate words, similarly as in Supplementary Note 4).

Supplementary Note 3. Robustness of the forecast effect.

Below, we show that the forecast effect holds without PCA, with different window sizes, when using *banded* ridge regression (87, 88) instead of ridge regression, when averaging instead of summing vectors within each TR, when matching the TR with the word onset instead of word offset, when accounting for low-level speech features and when testing for significance across windows at the single-subject level.

Replication with banded ridge regression In the main manuscript, we use ℓ_2-regularized ridge regression (as in e.g. (42)) followed by a hierarchical comparison of the brain scores: i.e. computing the brain score of the two sets of features (here, X vs. $X \oplus \widetilde{X}$) and then subtracting the

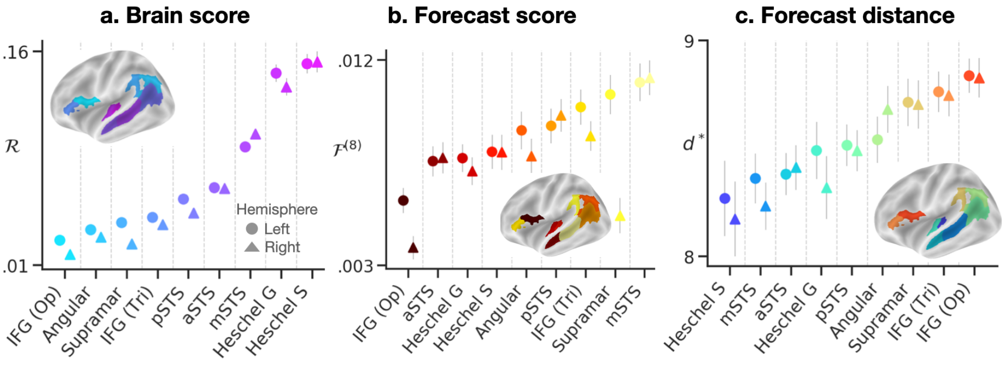

Supplementary Figure 1. Scores per region of interest. a-c. Brain scores (Figure 2**a**, Methods D), forecast scores (Figure 2**c**, Methods F) and forecast distance (Figure 2**e** Methods G) for nine regions of interests in both the left (circle) and right (triangle) hemispheres. Scores are averaged across voxels within each region of interest and across subjects. Error bars are the standard errors of the mean across subjects. Regions are ordered with respect to their average score in the left hemisphere.

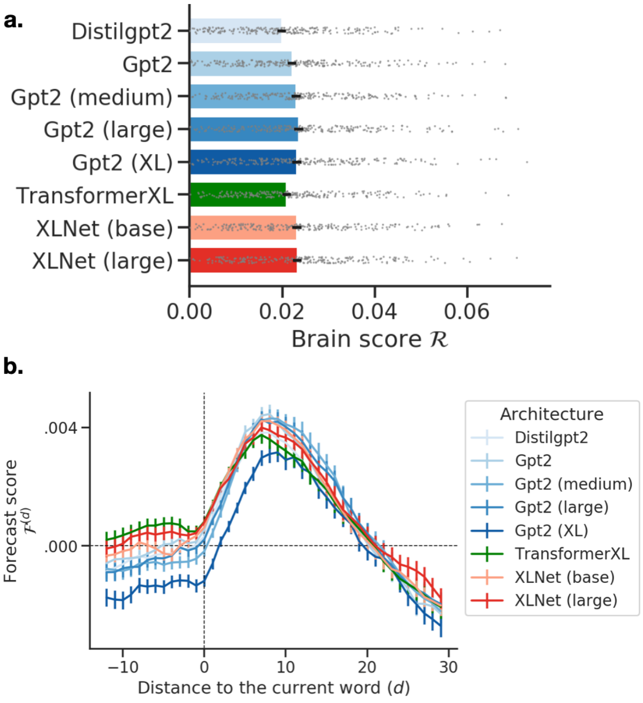

Supplementary Figure 2. Generalisation to other architectures. a. Brain scores (cf. Figure 1**b**, Methods D) of eight transformer models, based on XLNet (71), TransformerXL (86) and GPT-2 (2) architectures. We use the pre-trained models from Huggingface and proceed similarly as with GPT-2 (Methods C). Brain scores are averaged across voxels and subjects, error bars are the standard errors of the mean across subjects (n=304). **b.** Same as Figure 2**d** for the eight transformer architectures.

scores ($R[X \oplus \widetilde{X}] - R[X]$). To our knowledge, this approach is most conservative when it comes to assess the explained variance of the highest level: the explainable variance shared by two sets of features is by definition fully attributed to the lower-level feature set (i.e. X). Thus, in the worst case scenario, our method underestimates the variance specific to $\widetilde{X}$. This is what happens when the sliding window contains far off future words that are no longer relevant for prediction, and $R[X \oplus \widetilde{X}]$ becomes smaller than $R[X]$.

We replicate our results with banded ridge regression (87, 88) using the Himalaya (https://github.com/gallantlab/himalaya) package (88). Both X and $\widetilde{X}$ models are fitted simultaneously with a specific penalization term learnt for each submodel. We then evaluate the unique variance accounted for by each submodel by zeroing-out either X or $\widetilde{X}$ at test time, predicting Y, and computing Pearson's correlation between predicted and actual Y after zeroing out the specific features. We use the same cross-validation setting as in the paper.

Supplementary Figure 3**a** below displays the brain scores obtained with banded ridge when adding the window for each future word, and Supplementary Figure 3**b** shows the brains scores specifically attributed to the contextual words in $\widetilde{X}$ after zero-ing out X. We obtain similar results as in the original paper, but the forecast effect specific to $\widetilde{X}$ is higher than the one in the paper ($R''[\widetilde{X}]$ peaks at 0.027, while ($R[X \oplus \widetilde{X}] - R[X]$ peaks at 0.004).

Replication without PCA In the manuscript, we apply PCA to the GPT-2 features before applying the FIR and regression (Figure 8 and 9). We show in Supplementary Figure 3**c** that the forecast effect holds without applying PCA.

Replication without silent periods and with confounding variables In the main manuscript, we cut the TRs that do not contain words at the beginning of the stories, and do not add to the GPT-2 features confounding variables such as the phoneme rate and word rate. In Supplementary Figure 3**h**, we show that the results hold when the brain and forecast score are computed:

- When removing the empty TRs both at the beginning and end of the recordings (we thus cut the recordings between the first and last word of the story before fitting the ridge regression)
- When including the Word and Phoneme rates as confound variables. These are one-dimensional variables indicating the presence or absence of a word/phoneme.

Replication with different word aggregation in FIR In Supplementary Figure 3**d**, we show that results hold when averaging instead of summing vectors within each TR and when matching the TR with the word onset instead of word offset.

Testing for significance at the single-subject level In the main manuscript, we compare $R(X + X^{(i)})$ to $R(X)$ within each subject and then test the significance *across subjects* ($H_0 : R(X + X^{(d)}) < R(X)$). We show the results hold when testing for significance with a bootstrap test *across windows*, at the single-subject level ($H0 : R(X + X^{(d)}) < R(X + X^{(i)}), i \neq d$). Precisely, for each subject and each distance d, we compute $R(X + X^{(i)}), i \neq d$, with $X^{(i)}$ a sliding window randomly sampled from the stories. We repeat the procedure 1000 times and then estimate the probability of sampling $X^{(i)}$, such that $R(X + X^{(d)}) < R(X + X^{(i)})$. This results in a p-value for each subject and distance d, assessing the significance of $R(X + X^{(d)})$ being greater than $R(X + X^{(i)}), i \neq d$.

In Supplementary Figure 3**f-g**, we show that testing for significance at the single-subject level yields to similar conclusions as across subjects.

Effect of window size In the main manuscript, we use a fixed window size of seven words because it led to the best brain score when varying the length of the window (Supplementary Note 4). To further assess the impact of the window length on the forecast effect, we compute the forecast scores for different window sizes (from a size of 5 to 27 words). In Supplementary Figure 3**e**, we find that window length slightly but significantly affects the results. The distances maximizing the forecast scores are on average concentrated between 6 and 12 words, and brain scores are highest for a window of 7-9-11 words. The peak varies with the window length. This phenomenon is partly expected: words that are close to the current word likely carry relevant information (e.g. word n+1). Thus, for short window sizes, not including the closest words is expected to decrease the brain score. This confirms that the forecast result can be found regardless of the window size, and further suggests that forecasts are likely to be slightly longer-term than 8 words.

Supplementary Note 4. Controls with a growing window analysis.

Testing different window sizes In the previous paragraphs, we use a sliding forecast window with a *fixed* number of words in order to compare the brain scores of representations with the same dimensionality. Here, we test different window sizes by implementing a growing window analysis. Precisely, we build the forecast window $\tilde{X}^{(d)}$ by concatenating the d words succeeding the current word. The size of the window thus varies and d corresponds to both the number of words in the window, and the distance between the last word and the current word. We proceed similarly as in the main manuscript, build forecast window for different distances d and the corresponding forecast scores. As displayed in Supplementary Figure 4, the forecast score is maximal for a window of 8 future words ($d^* = 7.9 \pm 0.5$ on average across subjects), which is consistent with the previous results (Figure 2**c**, where $d^* = 8$).

Using random forecast representations We use the same growing window framework and check that adding a forecast window composed of random words does not improve the brain score (Supplementary Figure 4). Precisely, we randomly pick words out of all stories, concatenate the GPT-2 activations of random words to build the forecast windows $\tilde{X}^{(d)}$, and compute the corresponding forecast scores for different distances d. Supplementary Figure 4 shows that random forecast windows do *not* improve our ability to predict brain activity.

Using GPT-2 generations as forecast representations To what extent are the improvements in brain score due to (1) additional information about future words and/or (2) a different way to represent past words? To address this question, we repeat the same analysis with a forecast window input, not of the *true* future words, but with the words *generated* by GPT-2. Specifically, for each word w_k, we 1) input GPT-2 with its past context $w_0, \ldots w_k$, 2) generate future words $w'_{k+1}, \ldots w'_{k+n}$ using different decoding methods (greedy and sampling schemes), 3) extract the corresponding activations $X'_{k+1}, \ldots X'_{k+n}$, 4) build the growing windows from these activations and 5) compute their forecast scores. Thus, the brain signals, the current activations X_k and the activations of generated words $X'_{k+n} \ldots X'_{k+n}$ are all distinct transformations of the same past words $w_0, \ldots w_k$. Note that for step 2), we use Huggingface's sampling scheme with topk=50 and topp=0.95, do_sample=True, max_length=100. For the greedy scheme, we simply set do_sample to False, topp and topk to 1. (13)). The results show that a window made of *generated* words improves the brain score, although less so than a window made of the *true* words of the stories (Supplementary Figure 4), confirming that GPT-2 is an imperfect forecaster.

Supplementary Note 5. Contribution of each future word in the forecast effect.

In Figure 2**b**, we show that adding a sliding window containing future words improves our ability to predict brain activity. To interpret the impact of each word in this improvement, we launch a zero-out analysis. Precisely, we proceed as follows:

- At train time, we proceed similarly as in the main analysis (Figure 2**b**) and fit the regression using the current word embedding, concatenated to the sliding window.
- At test time, we zero out the features corresponding to all words after word k (i.e. we replace their embeddings by zeros).
- Finally, we report the Pearson correlation between predicted and actual brain data, when zeroing out words after word k.

This evaluates the importance of the words after word k in the prediction. We repeat the procedure for $k = 1$ to $k = 17$. Note that if the words-to-TR transform had been linear, this analysis would have been identical to an analysis of the coefficients.

We find that zeroing out future words triggers a clear drop in performance (Supplementary Figure 5). This demonstrates that each future word significantly contributes to the prediction in the ridge regression.

To further address this issue, we compute the brain scores when concatenating different continuations to the current word embedding. Specifically, we run the exact same analysis as Figure 2**b**, but replacing future words by either zeros or random continuations. These continuations are sensible phrases, of the same length as the true continuations, but randomly sampled from all stories. Supplementary Figure 6 below confirms that adding random continuations does not improve the brain scores.

Overall, Supplementary Figure 5 and 6 show that each future word up to ≈ 10 plays a significant role in the ridge regression.

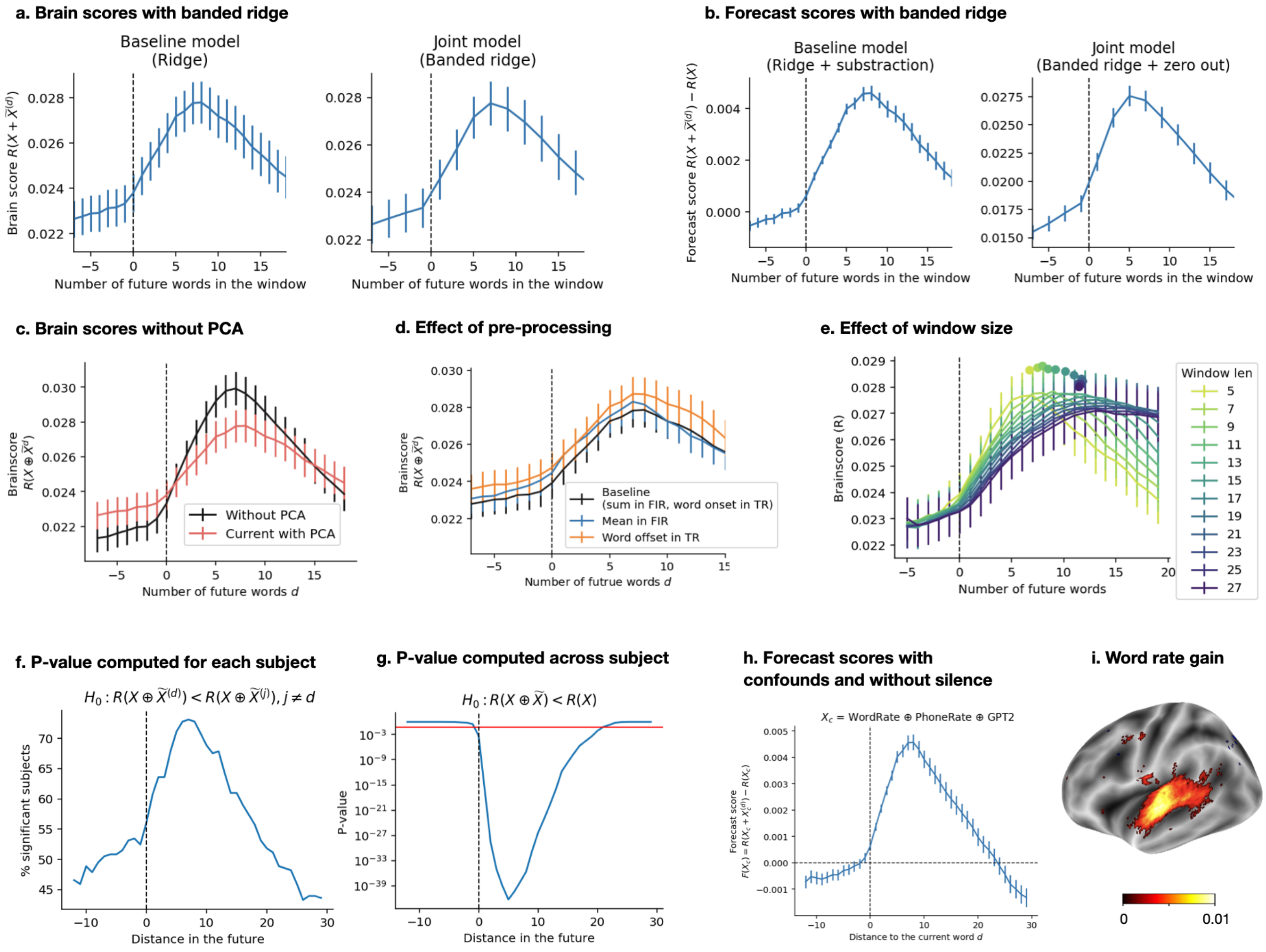

Supplementary Figure 3. Robustness of the forecast effect. a. Replication with banded regression. Brain scores computed with ridge regression (left, same as 2 and banded ridge regression (right) (87, 88). **b.** Forecast scores computed with ridge regression followed by subtraction (left, same as Figure 2**d**) and banded ridge regression followed by zero-out (right) (87, 88). In a banded ridge regression, a model is fitted using both X and $\widetilde{X}$ as input with regularization parameters specific to X and $\widetilde{X}$. We then evaluate the brain score accounted for by the context window $\widetilde{X}$ specifically by zero-ing out X at test time (and the present word in the window). **c.** Forecast scores without PCA. Brain scores when adding the sliding forecast window (Same as Figure 2**b**), but without applying PCA before fitting the ridge regression. **c.** Impact of pre-processing parameters. Brain scores when adding the sliding window for different distances d (same as Figure 2) (black), but averaging words within TR instead of summing them (blue) and matching the word offset with the TR boundary instead of the word onset (orange). **d.** Effect of window size. Brain scores when adding the forecast window (same as Figure 2**b**) computed with a sliding window of size 5 to 27 words. Average peaks across subjects are indicated with a dot. **f-g.** Significance of the forecast effect. In **f.**, the percentage of subjects with a significant bootstrap test for each distance d ($p < 0.05$). For each subject and each distance d, we compute $R(X + X^{(i)}), i \neq d$, with $X^{(i)}$ a sliding window randomly sampled from the stories. We repeat the procedure 1000 times and then estimate the probability of sampling $X^{(i)}$ such that $R(X + X^{(d)}) < R(X + X^{(i)})$, for each subject and distance d. In **g.**, the p-value computed with a one-sided Wilcoxon test across subjects, testing whether the sliding window improves the brain score $(R(X + X^{(d)}) > R(X))$. The red bar indicates the significance threshold ($p = 0.05$). **h.** Forecast scores with confounds and without silent periods. Forecast scores averaged across subjects and voxels (same as 2**b**) when (1) including two confounding variables (the word and phone rates) and (2) removing periods without words at the beginning and end of the recordings. The word and phone rates are one-dimensional variables indicating the absence/presence of a word and phoneme. **i.** Word rate gain. Gain in brain score when adding the word rate to the features of GPT-2, averaged across subjects (R[GPT2 ⊕ WordRate] - R[GPT2]). The WordRate is a one dimensional variable equal to one when there is a word, zero elsewhere. Only significant voxels are displayed (p<0.01 with a two-sided Wilcoxon test after FDR correction for multiple comparison). No PCA was performed.

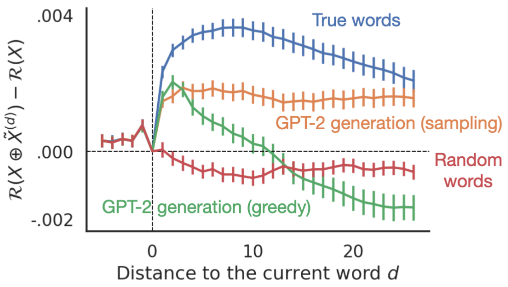

Supplementary Figure 4. Controls with a growing window analysis. Forecast scores for different types of forecast representations $\widetilde{X}$. Here, we use a growing window analysis: $\widetilde{X}^{(d)}$ is the concatenation of the activations of $|d|$ future ($d > 0$) or past ($d < 0$) words; the size of the window thus varies with the distance. The forecast score is the gain in brain score when concatenating the forecast window (cf. Eq. (3)). In blue, $\widetilde{X}$ is built out of the true words of the story. In red, $\widetilde{X}$ is built out of randomly picked words from all stories. In green and orange, $\widetilde{X}$ is built out of words generated by GPT-2. Precisely, GPT-2 is input with the current word and its previous context, and we use greedy (green) and sampling (orange) decoding schemes to generate a sequence of expected words. For simplicity, when $d < 0$, $\widetilde{X}$ is the concatenation of d the *true* past words. When $d > 0$, $\widetilde{X}$ is the concatenation of d future words (either true, generated or random words).

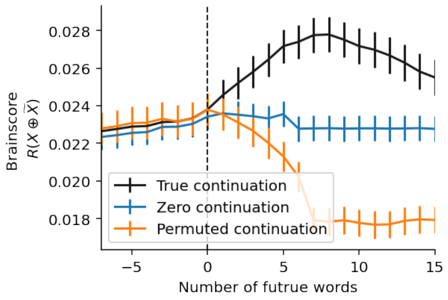

Supplementary Figure 6. Brain scores when adding different continuations. Same as Figure 2**b**, but true continuations (black) are replaced by zeros embeddings (blue) and random continuations sampled from all stories (orange). Random continuations are sensible phrases.

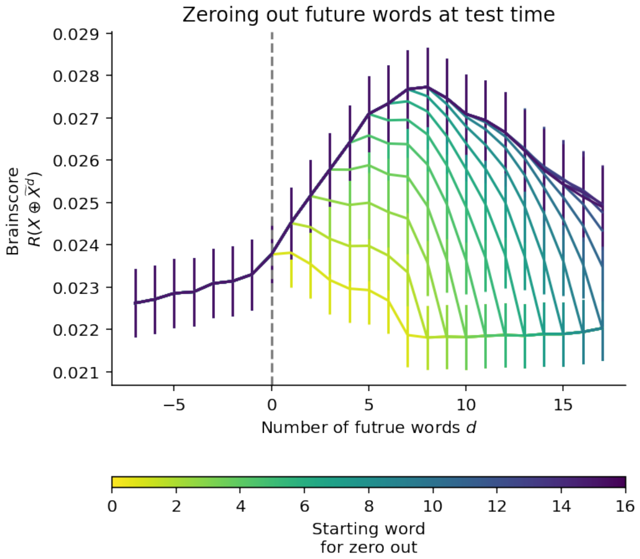

Supplementary Figure 5. Contribution of future words in the ridge regression. We proceed similarly as in Figure 2**b** and fit a ridge regression to predict the fMRI given X and the sliding window $\widetilde{X}^{(d)}$. Yet, at test time, we set to zero (or "zero-out") the dimensions corresponding to all words after word k. We then evaluate the prediction given the zeroed-out input (Pearson's correlation between predicted and true fMRI). On the x-axis, the last word that is not zeroed-out (k, i.e. all words > k are zeroed-out). On the y-axis, the corresponding Pearson correlation.

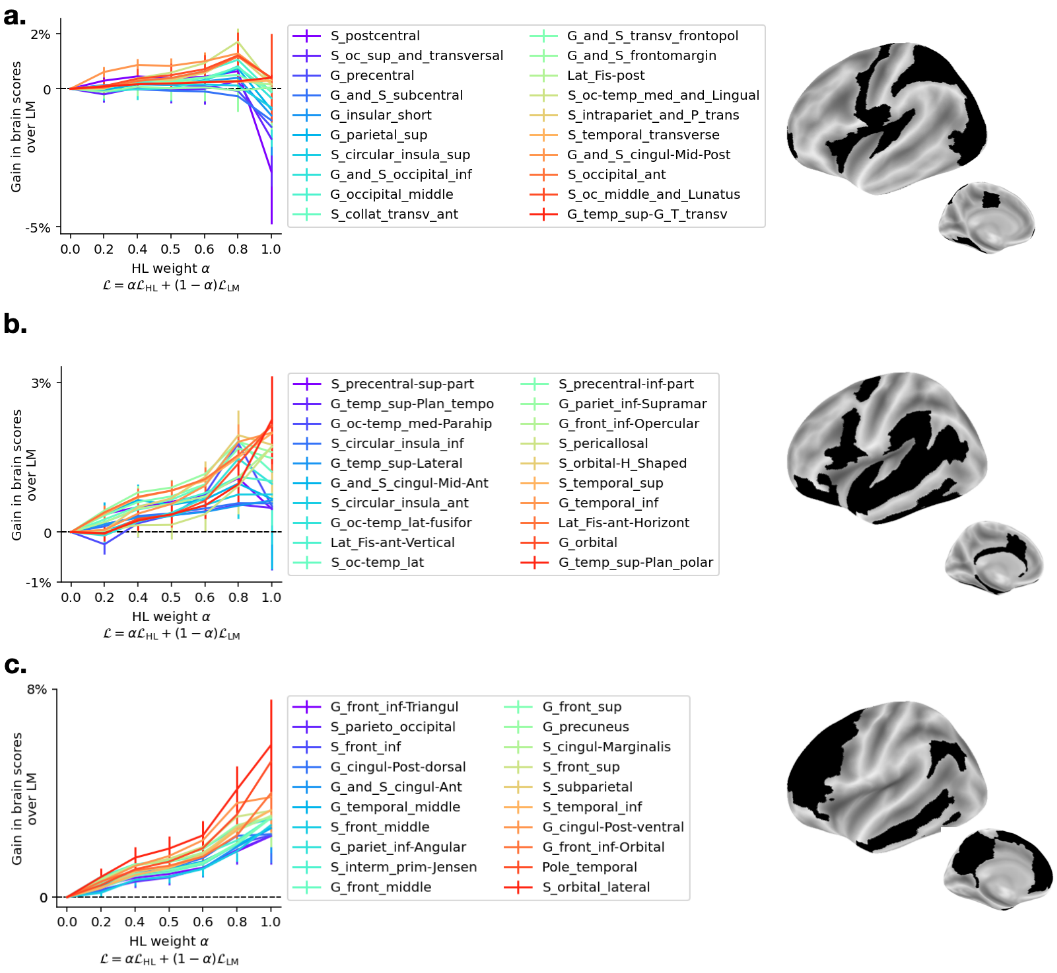

Supplementary Figure 7. Gain in brain scores when fine-tuning GPT-2 with a mixture of Language Modeling (LM) and High-Level prediction (HL). Gain in brain scores when adding the HL loss, compared to LM only as a function of the weight α_{HL} (Eq. (8)). Regions are grouped with respect to their gain, from negative or null improvement (**a.**) to high improvement (**c**). In black, the corresponding regions in the brain. Error bars are SEM across subjects. Brain scores were computed at the voxel-level and then averaged across voxels within 75 regions of interest using Destrieux's parcellation (82). We only display the 60 regions with highly significant brain scores ($p < 10^{-15}$ using a two-sided Wilcoxon test after FDR correction for multiple comparison across regions).

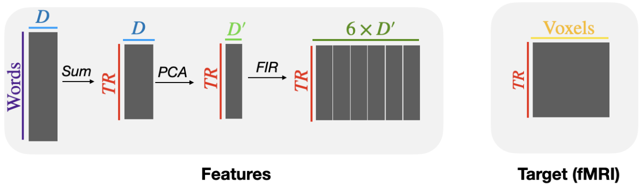

Supplementary Figure 8. Data pipeline *without* sliding window. Processing steps applied to the raw data of each subject before fitting the ridge regression. The ridge regression is then trained to predict the fMRI target (on the right) given the features (on the left) using a 5-folds cross-validation setting. D is the dimensionality of the language model, here $D = 768$. Words refers to the number of words in the audio recordings the subject listened to while being scanned. If the subject listened to more than one story, the audio recordings are concatenated and Words is the sum of the words of each story. TR is the number of the corresponding fMRI scans. D' is the dimensionality after PCA reduction, here D'=20. 6 is the number of delays used in the FIR.

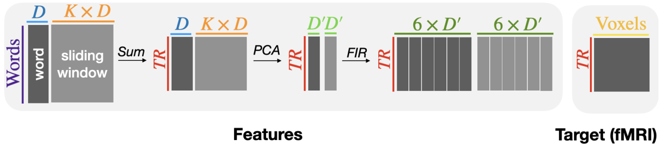

Supplementary Figure 9. Data pipeline *with* sliding window. Same as Figure 8, but we concatenate the sliding window to the current word (in orange and light grey). The sliding window contains the GPT-2 embeddings of past and/or future words. K is the number of words in the sliding window, here $K = 7$.

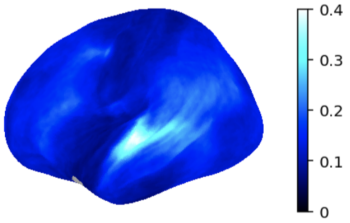

Supplementary Figure 10. Noise ceiling. Noise ceiling estimates averaged across subjects, for each voxels of the left hemisphere (Methods M).
