# Supplementary material for: Evidence of a predictive coding hierarchy in the human brain listening to speech
Source: Nat Hum Behav. 2023 Mar 2;7(3):430–41. doi: 10.1038/s41562-022-01516-2 (PMC10038805; doi:10.1038/s41562-022-01516-2)
Supplement: Supplementary file 2 — Reporting Summary [file 41562_2022_1516_MOESM2_ESM.pdf]

nature portfolio

Corresponding author(s): Charlotte Caucheteux

Last updated by author(s): Nov 25, 2022

Reporting Summary

Nature Portfolio wishes to improve the reproducibility of the work that we publish. This form provides structure for consistency and transparency in reporting. For further information on Nature Portfolio policies, see our Editorial Policies and the Editorial Policy Checklist.

Statistics

For all statistical analyses, confirm that the following items are present in the figure legend, table legend, main text, or Methods section.

n/a	Confirmed	
☐	☒	The exact sample size (*n*) for each experimental group/condition, given as a discrete number and unit of measurement
☐	☒	A statement on whether measurements were taken from distinct samples or whether the same sample was measured repeatedly
☐	☒	The statistical test(s) used AND whether they are one- or two-sided<br>*Only common tests should be described solely by name; describe more complex techniques in the Methods section.*
☐	☒	A description of all covariates tested
☐	☒	A description of any assumptions or corrections, such as tests of normality and adjustment for multiple comparisons
☐	☒	A full description of the statistical parameters including central tendency (e.g. means) or other basic estimates (e.g. regression coefficient) AND variation (e.g. standard deviation) or associated estimates of uncertainty (e.g. confidence intervals)
☐	☒	For null hypothesis testing, the test statistic (e.g. *F*, *t*, *r*) with confidence intervals, effect sizes, degrees of freedom and *P* value noted<br>*Give P values as exact values whenever suitable.*
☒	☐	For Bayesian analysis, information on the choice of priors and Markov chain Monte Carlo settings
☐	☒	For hierarchical and complex designs, identification of the appropriate level for tests and full reporting of outcomes
☐	☒	Estimates of effect sizes (e.g. Cohen's *d*, Pearson's *r*), indicating how they were calculated

Our web collection on statistics for biologists contains articles on many of the points above.

Software and code

Policy information about availability of computer code

Data collection	No data collection was performed
Data analysis	All analyses were performed using python (3.7) and scikit-learn. The fMRI data was analyzed using nilearn, MNE-Python, and freesurfer. The NLP algorithms were analyzed using the open source transformers library. Statistical significance was assessed using Scipy.

For manuscripts utilizing custom algorithms or software that are central to the research but not yet described in published literature, software must be made available to editors and reviewers. We strongly encourage code deposition in a community repository (e.g. GitHub). See the Nature Portfolio guidelines for submitting code & software for further information.

Data

Policy information about availability of data

All manuscripts must include a data availability statement. This statement should provide the following information, where applicable:

- Accession codes, unique identifiers, or web links for publicly available datasets
- A description of any restrictions on data availability
- For clinical datasets or third party data, please ensure that the statement adheres to our policy

The Narratives dataset (Nastase et al., 2020) is publicly available on OpenNeuro and Datalad platforms.

Field-specific reporting

Please select the one below that is the best fit for your research. If you are not sure, read the appropriate sections before making your selection.

☒ Life sciences ☐ Behavioural & social sciences ☐ Ecological, evolutionary & environmental sciences

For a reference copy of the document with all sections, see nature.com/documents/nr-reporting-summary-flat.pdf

Life sciences study design

All studies must disclose on these points even when the disclosure is negative.

Sample size	We studied all the subjects in the original dataset, n=345 (Nastase et al. 2020).
Data exclusions	As suggested in the original dataset, subjects were excluded because of noise or non-natural stimuli (Nastase et al. 2020). This results in a dataset of 304 subjects.
Replication	The experiment was successfully replicated on each of the 304 subject. Statistical significance is systematically assessed across subjects.
Randomization	For each subject, the stimuli (aggregated stories) were split into five folds. Then, a model was trained on four folds (80% of the words) and tested on the last fold (20% of held out words). The procedure was repeated for each possible split (five) and the results were averaged across the five test folds.
Blinding	For each subject, we blind 20% of the subject data. The model is trained on 80% and evaluated on the 20% left out data.

Reporting for specific materials, systems and methods

We require information from authors about some types of materials, experimental systems and methods used in many studies. Here, indicate whether each material, system or method listed is relevant to your study. If you are not sure if a list item applies to your research, read the appropriate section before selecting a response.

Materials & experimental systems

n/a	Involved in the study	
☒	☐	Antibodies
☒	☐	Eukaryotic cell lines
☒	☐	Palaeontology and archaeology
☒	☐	Animals and other organisms
☒	☐	Human research participants
☒	☐	Clinical data
☒	☐	Dual use research of concern

Methods

n/a	Involved in the study	
☒	☐	ChIP-seq
☒	☐	Flow cytometry
☒	☐	MRI-based neuroimaging
